# Fibro-fatty remodelling in arrhythmogenic cardiomyopathy

**DOI:** 10.1007/s00395-022-00929-4

**Published:** 2022-04-19

**Authors:** Arwa Kohela, Eva van Rooij

**Affiliations:** 1 Hubrecht Institute, Royal Netherlands Academy of Arts and Sciences (KNAW), Utrecht, The Netherlands; 2 Department of Cardiology, University Medical Center Utrecht, Utrecht, The Netherlands

**Keywords:** Arrhythmogenic cardiomyopathy, Fibro-fatty remodelling, Cellular origin, Pathways

## Abstract

Arrhythmogenic cardiomyopathy (AC) is an inherited disorder characterized by lethal arrhythmias and a risk to sudden cardiac death. A hallmark feature of AC is the progressive replacement of the ventricular myocardium with fibro-fatty tissue, which can act as an arrhythmogenic substrate further exacerbating cardiac dysfunction. Therefore, identifying the processes underlying this pathological remodelling would help understand AC pathogenesis and support the development of novel therapies. In this review, we summarize our knowledge on the different models designed to identify the cellular origin and molecular pathways underlying cardiac fibroblast and adipocyte cell differentiation in AC patients. We further outline future perspectives and how targeting the fibro-fatty remodelling process can contribute to novel AC therapeutics.

## Introduction

Arrhythmogenic cardiomyopathy (AC) is a genetic disorder that usually manifests with three main clinical phases: (I) the concealed phase, with minimal clinical symptoms, but with a risk of sudden cardiac death; (II) the electrical phase, with symptomatic ventricular arrhythmias in the form of ventricular tachycardias or ventricular fibrillation, palpitations, and syncope, together with structural abnormalities; and (III) the end-stage phase, with severe structural changes, ventricular dilation, and dysfunction which can progress to heart failure [4, 16, 58]. The prevalence of AC is 1:2000 to 1:5000 depending on the population, and has been found to mainly affect young individuals and athletes [17, 77]. Despite the complex disease aetiology, with multiple risk factors contributing to disease pathogenesis, genetic predisposition remains the main underlying cause of AC. A familial background has been associated with about 60% of AC cases with an autosomal dominant mode of inheritance [36, 45]. Most of AC-related mutations (~ 54% of all AC cases) have been linked to genes encoding components of desmosomes; intercellular junctions expressed by cardiac muscle and epithelial tissue. These include plakophilin-2 (*PKP2*), desmoplakin (*DSP*), desmocollin-2 (*DSC2*), desmoglein-2 (*DSG2*) and plakoglobin (*JUP*). However, mutations in the non-desmosomal genes transmembrane protein 43 (*TMEM43*), phospholamban (*PLN*), transforming growth factor beta 3 (*TGFB3*), desmin (*DES*), titin (*TTN*), αT-catenin (*CTNNA3*), and lamin A/C (*LMNA*), have been also linked to AC in ~ 5% of the cases [36, 45].

**Fig. 1:**
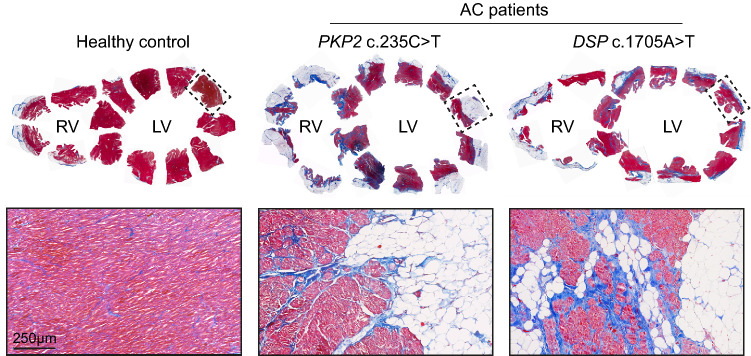
Cardiac fibro-fatty tissue remodelling in AC patients. Masson trichrome staining of explanted human hearts from a healthy control and AC patients showing cardiomyocytes in red, fibrosis in blue and adipocytes in white. Upper panels show full ventricular sections and insets indicate slices where higher magnification images were obtained. Lower panels show fibro-fatty tissue replacement within myocardial regions of the corresponding ventricular slices. RV, right ventricle; LV, left ventricle. Adapted from Sepehrkhouy et al*.* [69] and Kohela et al*.* [43]

The most striking histopathological feature of AC is the loss of ventricular myocardium, possibly due to cardiomyocyte atrophy and apoptosis, and its replacement with fibro-fatty tissue. Fibro-fatty tissue deposition typically extends from the epicardium towards the endocardium and is usually associated with inflammatory infiltrates [5, 16] (Fig. 1). As the disease progresses, fibro-fatty tissue can act as a substrate to aggravate arrhythmias further hindering proper ventricular function [20, 76].

Many cellular and animal models of AC are available to help understand different aspects of AC pathogenesis such as ventricular dysfunction, cardiomyocyte death and inflammation [3, 33]. However, recapitulating and studying the fibro-fatty infiltration process characteristic of AC has been limited due to the natural resistance of most experimental animal models to cardiac adipose tissue development [82]. In this review, we summarize our understanding on the cellular origin and mechanisms of fibroblast and fat cell differentiation in AC.

## Cellular origin

In recent years, there has been considerable efforts to elucidate the cellular effectors contributing to the replacement of ventricular myocardial tissue with extensive fibro-fatty infiltrates in AC patients. Studies implemented different models of dysfunctional desmosomes due to mutations or gene knockouts. These included mouse and zebrafish as well as cellular models of AC such as human-induced pluripotent stem cell (hiPSC)-derived cardiac cultures and explanted human AC hearts. So far, cell types which have been proposed to act as a source of fibro-fatty tissue in AC are cardiomyocytes, cardiac progenitor cells and epicardial cells. These studies are summarized in Table 1 and discussed below.

**Table 1:** Summary of studies investigating fibro-fatty remodelling in AC

Proposed cellular origin	Models used	Main findings related to fibro-fatty remodelling	Ref #
Cardiomyocytes	Explanted human AC heart	Cardiomyocytes adjacent to fibro-fatty tissue of an explanted human AC heart contain lipids and stain positive for vimentin.	[18]
	Explanted human AC heart	Cardiomyocytes of an explanted human AC heart contain lipids.	[30]
	hiPSC-cardiomyocytes	*PKP2* mutant hiPSC CMs undergo lipogenesis following adipogenic stimulation due to reduced Wnt signalling.	[10]
	hiPSC-cardiomyocytes	*PKP2* mutant hiPSC-cardiomyocytes undergo lipogenesis following adipogenic stimulation due to activated PPARγ signalling.	[42]
	hiPSC-cardiomyocytes	*PKP2* mutant hiPSC-cardiomyocytes undergo lipogenesis following adipogenic stimulation.	[56]
	Cardiomyocyte-specific *Dsp*^−/−^ mice	Some adipocytes at the sub-epicardium in *Dsp*^−/−^ mice originate from an MLC2v^+^ cardiomyocyte lineage.	[55]
Isl1^+^ Wt1^+^ myo-adipo progenitors	Isl1/Wt1 lineage traced mice	A common cardiomyocyte and adipocyte Isl1^+^/Wt1^+^ progenitor underlies adipogenesis in AC.	[24]
Isl1^+^ Mef2c^+^ progenitors	*Dsp*^±^ lineage-traced mice	Isl1^+^ Mef2c^+^ second heart field progenitors give rise to most adipocytes in *Dsp*^±^ mice due to PKG nuclear translocation and WNT inhibition.	[54]
c-Kit^+^ Sca1^+^ progenitors	Transgenic mice overexpressing mutant PKG (PKG^Trg^)	c-Kit^+^ Sca1^+^ progenitors isolated from PKG^Trg^ mice undergo lipogenesis upon adipogenic stimulation due to WNT signalling inhibition.	[53]
Fibro-adipocyte progenitors (FAPs)	Human and mouse isolated FAPs and *Dsp*^±^ mice	Cardiac FAPs are a PDGFRA + progenitor cell population which expresses COL1A1 or CEBPA and can differentiate into fibroblasts or adipocytes, respectively. 40% of adipocytes in *Dsp*^±^ mice arise from FAPs via WNT signalling inhibition.	[52]
	Transgenic mice overexpressing mutant DSG2 (DSG2^mu^)	PDGFRA^+^ HIC1^+^ FAPs give rise to fibroblasts and adipocytes in DSG2^mu^ mice.	[71]
Mesenchymal stromal cells (MSCs)	Explanted and bioptic samples from human AC and control hearts	Adipocytes in explanted human AC hearts express CD29 and CD105 indicating their mesenchymal origin. MSCs isolated from AC patients subjected to adipogenic stimuli display increased lipogenesis and adipogenic marker expression due to WNT pathway suppression.	[72]
Epicardial cells	Neonatal rat epicardial explants	*Pkp2* suppression in neonatal rat epicardial explants promotes their proliferation, migration, lipogenesis and cellular differentiation into α-SMA^+^ cells.	[60]
	Epicardial-specific *Dsp*^±^ mice	Fibroblasts in epicardium-specific *Dsp*^±^ mice arise via epicardial EMT through the expression of paracrine factors such as TGFβ1 and FGF.	[85]
	hiPSC-epicardial cells and explanted hearts from AC patients	hiPSC-epicardial cells undergo spontaneous fibro-fatty cellular differentiation upon desmosomal gene suppression due to enhanced EMT mediated by TFAP2A. Explanted human AC hearts display epicardial thickening, activation through WT1 expression, and TFAP2A induction in the sub-epicardial mesenchyme.	[43]

### Cardiomyocytes

Cardiomyocyte transdifferentiation into adipocytes during AC progression was first suggested based on the morphological examination of the ventricular myocardium of a female transplant patient [18]. Cardiomyocytes adjacent to fibro-fatty tissue contained sarcoplasmic vacuoles with a lipidic nature that highly resembled pre-adipocytes [18]. The authors therefore suggested a cardiomyocyte-to-adipocyte switch, as some of these cells also stained positive for vimentin, a marker expressed by adipocytes [18]. However, vimentin is a mesenchymal marker which is not exclusively expressed by adipocytes [19]. The presence of intracellular lipid droplets in cardiomyocytes has been also described in biopsied myocardial tissue of another AC patient [30]. These lipids were found in degenerating cardiomyocytes and were often discharged into the interstitial space upon cell membrane dissociation [30]. Despite the informative ultrastructural examination of explanted AC hearts, these studies were based on the histological observation of single cases.

In an in vivo setting, one report could show that some adipocytes arising in a *Dsp* knockout mouse model located at the sub-epicardium, but not at the midwall, originate from a cardiomyocyte lineage labelled by MLC2v [55]. Presence of a common “cardiomyocyte-adipocyte progenitor” cell population in normal hearts was further proposed in a study by Dorn et al*.* [24]. The authors described an Isl1^+^/Wt1^+^ progenitor cell population in normal hearts, which under different stimuli primes towards a myocytic or adipocytic fate, and hence could potentially contribute to adipocyte differentiation in AC [24].

Due to difficulties with recapitulating the fibro-fatty phenomenon in many AC mouse models, the use of hiPSCs presented an alternative tool to study the cardiomyocyte transdifferentiation hypothesis in vitro. In three subsequent reports, hiPSC-derived cardiomyocytes (hiPSC-CMs) generated from AC patients demonstrated several AC features such as reduced densities of desmosomal proteins and electrical instabilities [10, 42, 56]. Additionally, exposure of hiPSC-CMs to adipogenic stimuli induced lipid droplet accumulation, which suggested an underlying predisposition to adipocytic differentiation in AC [10, 42, 56]. However, the exposure of cells to an adipogenic environment is rather artificial and does not mimic human disease, hence the cardiomyocyte-to-adipocyte transdifferentiation theory requires further investigation.

### Cardiac progenitor cells

The presence of unique progenitor cell populations in the heart with a multipotent differentiation potential has gained much attention in the recent years. Different reports have suggested that during AC disease progression, resident cardiac progenitors can differentiate into fibroblasts, adipocytes, or both. Below, we discuss the different populations proposed.

#### Isl1^+^ Mef2c^+^ progenitors

In 2009, the group of A. J. Marian used lineage tracing to monitor the origin of adipocytes in *Dsp*-deficient mice [54]. Three cardiac lineage promoters were used: Nkx2.5 which labels descendants of first and second heart field progenitors as well as epicardial cells, Mef2c which labels descendants of only second heart field progenitors, and Myh6 which labels cardiomyocytes [54]. Most adipocytes in the *Dsp*-deficient mice were shown to derive from the Nkx2.5 and Mef2c lineage, but not from the Myh6 lineage, indicating that adipocytes in AC hearts can possibly arise from second heart field progenitors expressing Isl1 and Mef2c [54]. This has been suggested to occur due to PKG translocation to the nucleus which leads to suppressed WNT signalling and hence enhanced adipogenic differentiation in AC hearts [54]. This study demonstrates the possible contribution of second heart field progenitors to adipocytes arising in the right ventricle. However, presence of biventricular and left-dominant forms of AC argues against a second heart field origin of adipocytes.

#### c-Kit^+^ Sca1^+^ progenitors

To follow up on the role of PKG nuclear translocation and its role in AC pathogenesis, the same group further investigated the relation between PKG, WNT signalling and adipogenesis [53]. The authors generated transgenic mice overexpressing wild-type (PKG^WT^) or truncated PKG (PKG^TR^) as well as PKG null mice (PKG^−/−^) [53]. PKG^TR^ mice showed reduced membrane localization and binding to DSP and DSG2 [53]. Furthermore, PKG was found to be expressed in a progenitor cell population expressing c-Kit and Sca1, which, upon adipogenic stimulation, could undergo lipogenesis in vitro [53]. This effect was shown to be mediated through suppressed WNT signalling and reversed through WNT signalling activation [53]. Furthermore, c-Kit^+^ Sca1^+^ progenitors isolated from PKG^−/−^ embryos were resistant to adipogenesis and exhibited increased levels of WNT signalling activation, further suggesting the potential of c-Kit^+^ Sca1^+^ progenitors to undergo adipogenic differentiation [53]. However, the extremely low abundance of c-Kit^+^ Sca1^+^ cells in the heart, and emerging reports arguing against their pluripotent potential [75], limit their ability to act as a main source of adipocytes in AC.

#### Fibro-adipocyte progenitors

Fibro-adipocyte progenitors (FAPs) were first described in skeletal muscle as a quiescent population of cells which can rapidly proliferate and contribute to adipocyte and fibroblast differentiation after muscle injury [40, 78]. In the heart, Marian’s group could further identify a similar PDGFRA^+^ cell population with bipotential towards fibroblast and adipocyte differentiation [52]. Isolation of FAPs from human and mouse hearts showed that these cells expressed COL1A1 or CEBPA, which allowed to label them as fibroblast or adipocyte progenitors, respectively [52]. In a *Dsp*-deficient mouse model, the authors could show that ~ 40% of cardiac adipocytes originated from FAPs, indicating the contribution of FAPs, as well as other cell types, to adipogenesis in AC [52]. Corroborating their previous AC studies, the adipogenic potential of FAPs was found to be mediated through WNT signalling suppression, which when re-activated reduced adipogenesis in vitro [52]. Although the described FAPs population was selected based on the exclusion of the fibroblast markers THY1 and DDR2, most of these cells still expressed COL1A1, which is often depicted as an activated myofibroblast marker. Furthermore, interestingly, DSP was only expressed in the adipogenic, and not fibrogenic, subsets of FAPs, which made it difficult to trace the fibrogenic potential of *Dsp*-deficient FAPs [52].

Later in 2020, another study further described the role FAPs in models of myocardial infarction (MI) and AC [71]. The authors described FAPs as a multipotent resident progenitor cell population expressing PDGFRA and the progenitor cell markers SCA1 and HIC1 [71]. In response to MI, these cells were activated generating fibroblasts which contributed to scar tissue formation at the injury area [71]. In an AC mouse model overexpressing mutant DSG2, cells derived from the PDGFRA^+^ or HIC1^+^ lineage contributed to both fibroblast and adipocyte differentiation [71]. A similar phenotype was observed in *Hic1* knockout hearts [71]. However, in this study, it was unclear how different injury models triggered fibrogenesis only or fibro-adipogenesis following HIC1 suppression.

#### Mesenchymal stromal cells

Cardiac mesenchymal stem/stromal cells (MSCs) are multipotent progenitors of epicardial origin, which are important for the mechanical support of tissues by providing extracellular matrix and paracrine signals [63]. The contribution of cardiac resident MSCs to scar tissue formation was first reported in a model of MI [9]. The authors could show that CD44^+^ MSCs act as precursor cells which generate scar tissue fibroblasts upon injury [9]. In vitro, these cells expressed both stem cell and fibroblast markers and retained a self-renewal and multipotent capability when cultured in different lineage-induction media [9].

In the context of AC, a study by Sommariva et al*.* showed their potential to also differentiate into adipocytes [72]. In hearts of AC patients, adipocytes labelled by the mature adipocyte marker PLIN1 also stained positive for the mesenchymal markers CD29 and CD105, suggesting their mesenchymal origin [72]. Of note, the authors occasionally found rare populations of c-Kit^+^ adipocytes suggesting a possible, but limited, contribution of c-Kit^+^ progenitors to adipogenesis [72]*.* MSCs isolated from AC patients expressed higher levels of adipogenic markers and were more prone to undergo lipogenesis than control MSCs when subjected to adipogenic stimuli [72]. This effect was reduced following WNT pathway activation or PKP2 overexpression in patient MSCs [72]. Due to their high abundance in the heart and plasticity to differentiate into various mesenchymal lineages, MSCs present strong candidates to adipogenesis in AC. However, the isolation of these cells relies on their plastic adherence after digestion and their expression of mesenchymal markers (CD90, CD29, CD105, CD44, CD73) [66]. These properties are not specific for MSCs, but also found in other stromal cells such as fibroblasts [28, 66]. Furthermore, MSCs were isolated from patient auricles and not ventricles which show most fibro-fatty remodelling. In addition to their reported adipogenic potential in this study, it would be interesting to investigate the fibrogenic potential of these MSCs as well, owing to their previously described role in scar tissue formation following ischemic injury [9].

### Epicardial cells

The epicardium is the outermost layer of the heart composed of mesothelial cells that mainly remain quiescent in adult hearts. During development and after disease, epicardial cells can undergo epithelial-to-mesenchymal transition (EMT) giving rise to different cardiac cell populations [34, 70]. Given their multipotent cell potential [34, 70], the sub-epicardial predominance of fibro-fatty infiltrates in AC patients [45, 76], and the high epicardial expression of desmosomal genes [43, 60], epicardial cells have been also suggested as candidates to originate fibro-fatty tissue.

In the normal murine heart, fat tissue is often confined at a specific region named the atrial–ventricular (AV) groove [82]. A study by Yamaguchi et al*.* demonstrated that the AV groove fat originates from the epicardium through the EMT and PPARγ pathway activation [82]. Later, using lineage tracing on ischemic injury models, adipocytes emerging at the peri-infarct region following MI were shown to also partially derive from the adult epicardium [51, 86]. Additionally, epicardial-to-fibroblast differentiation due to adult epicardial EMT has been also suggested in models of MI [26, 68, 80].

Interestingly, in studies on atrial fibrillation, which is characterized by the extensive atrial remodelling with fibro-fatty tissue, fibroblasts and adipocytes were shown to arise from the epicardium [74]. This appeared to be due to a pre-programmed state of subsets of adult epicardial-derived cells (EPDCs) towards either fibroblast or adipocyte cell fates, which when activated undergo fibro-fatty differentiation and infiltration into the diseased atria [74].

In the context of AC, Matthes et al*.* were the first to show that epicardial explants from neonatal rat hearts express PKP2, which when silenced promotes cellular proliferation, migration, lipogenesis and cellular differentiation into α-SMA^+^ cells [60]. These data suggested an important role of the desmosome in maintaining the mechanical integrity of epicardial cells, which when lost could potentially promote cellular differentiation.

In addition to the previously discussed studies from Marian’s group suggesting the contribution of different progenitors to fibro-fatty differentiation [52–54], they could recently also demonstrate the role of the epicardium in AC pathogenesis [85]. Using a reporter mouse model carrying an inducible epicardial-specific *Dsp* deletion, the authors could show the epicardial origin of fibroblasts [85]. These cells were shown to express paracrine factors such as TGFβ1 and FGF, which mediate EMT as well as apoptosis, arrhythmias and cardiac dysfunction [85].

In a recently published report, our group further underscored the role of the epicardium in fibro-fatty remodelling. [43]. In this study, we made use of hiPSC-derived cardiac cultures generated from AC patients, their isogenic controls and healthy donors to study AC pathogenesis in vitro. We could show that hiPSC-derived epicardial cells undergo EMT and spontaneous fibro-fatty cellular differentiation upon desmosomal gene suppression, due to either intrinsic mutations or targeting siRNAs. Using single cell RNA sequencing, we identified transcription factor TFAP2A to mediate this process by enhancing EMT signalling in the diseased cells. Furthermore, we observed that human AC hearts display increased epicardial thickening and WT1 expression indicative of epicardial activation. Additionally, cells located at the sub-epicardial mesenchyme stained positive for WT1 and TFAP2A, which further suggested that an epicardial-derived subset of cells underlies the fibro-fatty phenotype.

### Other potential sources of fibroblasts

Various studies have tried elucidating the cellular origin of fibroblasts contributing to scar tissue formation in different forms of cardiovascular disease. Although not particularly studied in AC, these identified fibroblast progenitor populations might also potentially contribute to fibrosis in AC patients and hence are also discussed below.

All resident fibroblasts in the adult heart derive from the epicardium during development and remain quiescent under homeostatic conditions [31]. Several reports could demonstrate the presence of cardiac fibroblast progenitor cell populations which upon injury can contribute to the pathological fibroblast differentiation. In one study, an adult stem cell population labelled by PW1 was found to contribute to fibrotic remodelling after MI [83]. PW1^+^ cells were mainly found near infarct areas of human and mouse ischemic hearts and could give rise to fibroblasts labelled by FSP1 and α-SMA [83]. In another study, peri-vascular precursors, labelled by GLI1 expression, were also shown to contribute to fibrosis after injury in different organs including the heart [44]. Lineage tracing experiments showed that 60% of infarct fibroblasts arise from GLI1 + vascular progenitors [44].

Bone marrow-derived cells have been also suggested to differentiate into cardiac fibroblasts, although the contribution seemed to vary between different cardiac disorders. In different studies, mice were transplanted with genetically labelled or sex-mismatched bone marrow cells which were shown to significantly contribute to scar tissue fibroblasts after MI [61, 79] and in dilated cardiomyopathy [15].

Another study described a circulating population of leukocytes termed fibrocytes as a source of injury fibroblasts in models of angiotensin-II-induced cardiac hypertrophy [38] and ischemic cardiomyopathy [39]. These cells normally express both hematopoietic markers (CD34, CD45) and fibroblast markers (procollagen I, vimentin) [48]. Upon stimulation, fibrocytes are recruited to the injury site where they adopt a myofibroblast phenotype expressing α-SMA and produce collagen [48].

In contrast to these reports, other lineage-tracing studies could show that scar tissue fibroblasts mainly originate from pre-existing resident epicardium-derived fibroblasts and not from bone marrow or stem cell populations. This was observed in models of pressure overload [1, 62] and MI [29, 41, 67, 84].

Despite the potential of the described reports to identify the origin of fibroblasts in different cardiac disorders, it is unknown whether these findings also apply to AC where fibroblasts infiltrate massive regions of adipose tissue.

## Pathways implicated

AC is mainly considered a disease of cardiac desmosomes [21]. These multiprotein complexes are required for maintaining both mechanical and electrical signals throughout the heart [21]. The main pathways found to be potentially implicated in fibro-fatty remodelling in AC due to desmosomal dysregulation are WNT, Hippo, TGFβ and PPARγ signalling pathways.

### WNT pathway

Canonical WNT signalling is the most commonly accepted pathway involved in AC pathogenesis. β-catenin, together with PKG, also known as γ-catenin, acts to link cadherin proteins to the actin cytoskeleton [88]. In response to WNT ligands, intracellular β-catenin levels are stabilized leading to its translocation to the nucleus, where it activates target genes via binding to TCF/LEF1 transcription factors [88]. Since PKG functions as a constituent of the desmosome and has been also shown to inhibit the transcriptional activity of β-catenin [64, 88], WNT signalling has been suggested to be implicated in AC pathogenesis.

Aberrant WNT signalling in AC was first reported by Garcia-Gras et al. in 2006 [32]. The authors showed that loss of DSP expression in cultured atrial myocytes or in mice leads to the nuclear translocation of PKG, where it competes with β-catenin for binding to TCF/LEF1 and therefore inhibits β-catenin-mediated WNT signalling [32]. Since WNT signalling inhibition is known to promote adipogenesis [65], the authors suggested PKG nuclear translocation as an underlying mechanism to adipocyte differentiation in AC [32]. Reduced WNT signalling activity has been further demonstrated in other AC mouse models overexpressing mutant forms of *Jup* [53] and *Dsg2* [8] and in *Dsp*-deficient zebrafish [35]. However, conflicting to these data, cardio-restricted *Jup* knockout was shown to increase β-catenin stabilization and TCF/LEF transcriptional activity, which was suggested to underlie a cardiac hypertrophic phenotype in these mice with no signs of cardiac adipogenesis [47]. In another cardiac-specific *Jup* knockout mouse model, WNT signalling was shown to be unaltered [46]. In addition to its involvement in adipogenic remodelling, WNT signalling has been also linked to cardiomyocyte apoptosis and electrical instabilities [2, 37], suggesting a broader role for WNT signalling in AC pathogenesis. However, further investigation is needed to understand the causal mechanisms underlying WNT pathway dysregulation in AC patients.

### Hippo pathway

The Hippo pathway is well known for its roles in regulating cellular proliferation and apoptosis, thereby controlling growth of tissues [81]. Particularly in AC, a study showed that in human AC hearts, as well as in cell culture and mouse models of AC, Hippo signalling was activated [13]. This led to the phosphorylation of downstream Hippo molecules including the Hippo-effector molecule Yes-associated protein (YAP), which was found to interact with β-catenin and PKG, ultimately enhancing the adipogenic phenotype [13].

### TGFβ pathway

TGFβ signalling is key in regulating cardiac fibrosis and hence has been linked with fibrotic remodelling in AC [23, 49]. The association of TGFβ signalling with AC was first demonstrated by the identification of mutations in the UTR regions of TGFβ3 in two familial cases of AC, which were shown to increase the activity of TGFβ by twofold [7]. Furthermore, in a *Jup* knockout mouse model, which showed massive fibrosis and no alterations in WNT signalling, TGFβ signalling was significantly induced [46]. The authors suggested that the elevated TGFβ activity could be contributed to an increase in myocardial wall stress as a result of desmosomal loss [46]. Interestingly, the increase in TGFβ activity has recently also been validated in plasma collected from AC patients, which coincided with an increase in fibrotic markers in endomyocardial biopsies [57]. In this study, the authors found that MSCs isolated from AC patients were more prone to fibrotic differentiation in response to TGFβ1 treatment compared to healthy donors [57]. In another study, knockdown of *Pkp2* in cardiomyocytes induced a TGFβ-mediated induction of pro-fibrotic genes [27]. This effect was corroborated in tissues isolated from *Pkp2* and *Dsp*-deficient mouse and zebrafish models [27, 35]. In addition to its role in fibrotic differentiation, TGFβ is known for its crucial roles in promoting EMT signalling in the heart which can precede fibrotic remodelling [6, 25]. Recently, we could show that hiPSC-epicardial cells and primary human atrial EPDCs undergo excessive EMT in response to TGFβ1 treatment or desmosomal suppression [43]. This has been further corroborated in an epicardial-specific *Dsp* knockout mouse model which demonstrated an epicardial-derived origin of fibroblasts due to enhanced EMT and TGFβ signalling [85]. However, the exact link between AC-related mutations and EMT remains to be elucidated.

### PPARγ pathway

PPARγ is a nuclear receptor that functions as a master regulator of lipid uptake and adipogenesis [11]. Induction of PPARγ signalling together with increased lipogenesis have been described in different models of AC. These include *Dsp-* and *Pkp2*-deficient cardiomyocytes [24, 32, 42] and epicardial cells [43] as well as human AC hearts [22]. The exact mechanisms by which desmosomal dysregulation alters PPARγ signalling are not fully understood. However, one possible link could be the negative regulation of PPARγ by β-catenin [50], which is widely suggested to be inhibited in AC [8, 32, 35, 53].

## Concluding remarks

**Fig. 2:**
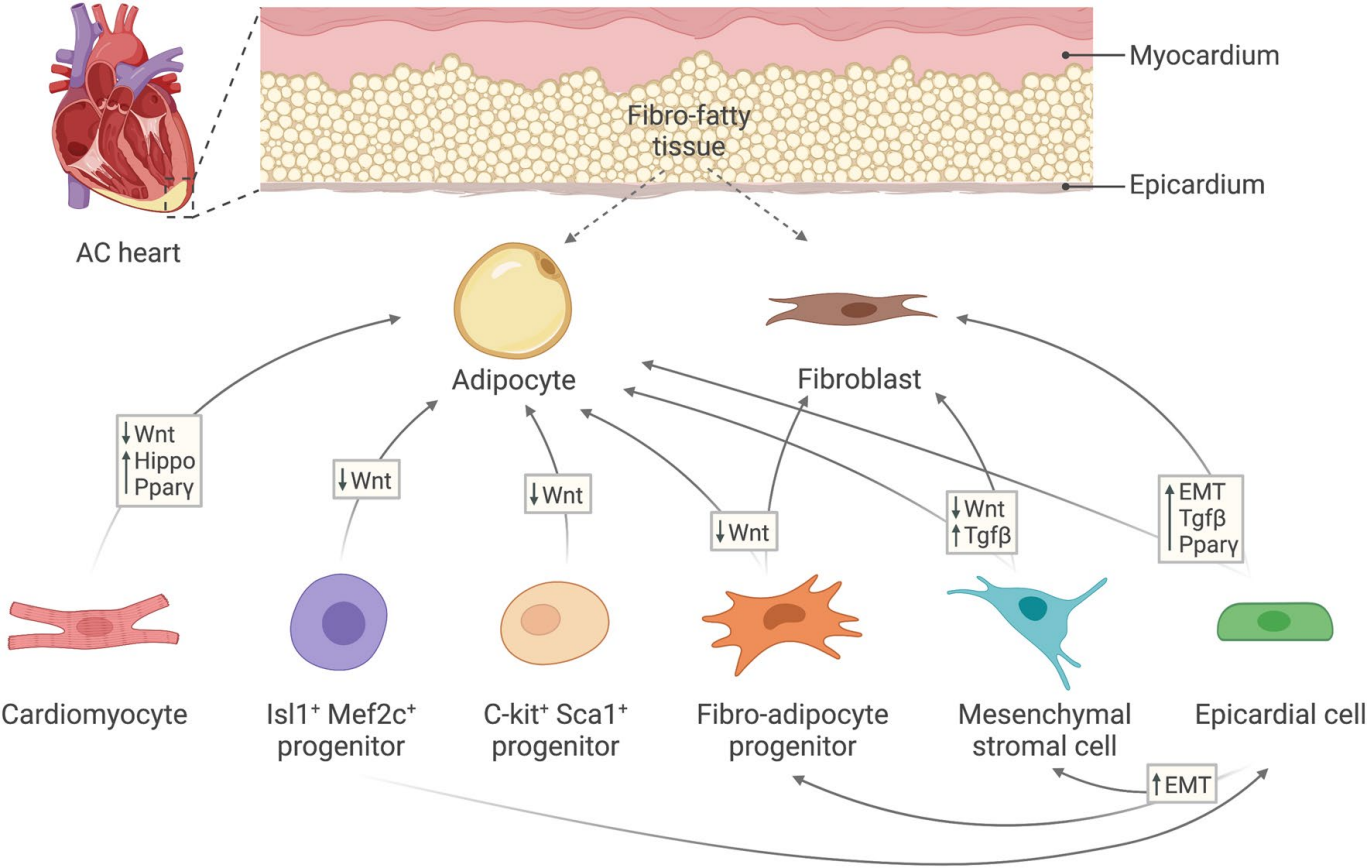
Schematic of the different cellular populations and pathways reported to underlie fibro-fatty remodelling in AC. Fibro-fatty tissue typically extends from the epicardium towards the myocardium in AC hearts. Depicted arrows indicate the potential of each cell population to differentiate into another and the proposed pathways or processes underlying this transition. *AC* arrhythmogenic cardiomyopathy; *Wnt* Wingless-related integration site; *Pparγ* peroxisome proliferator–activated receptor gamma; *Tgfβ* transforming growth factor beta; *EMT* epithelial-to-mesenchymal transition

AC is a multifaceted and progressive disorder. Its pathogenesis usually initially manifests with electrophysiological instabilities which can lead to lethal ventricular arrhythmias [4]. As the disease develops, fibro-fatty remodelling progressively intervenes, which can act as an arrhythmogenic substrate further hindering cardiac conductivity [20, 76]. In this review, we discussed the possible cellular origins and mechanisms which can underlie fibro-fatty tissue deposition in AC hearts (Fig. 2). However, whether the emergence of fibro-fatty tissue is a direct cause of AC-related mutations, a consequence to cardiomyocyte death and physiological instabilities, or possibly both, remains an open question.

As previously outlined, cardiomyocyte-to-fibroblast/adipocyte transdifferentiation is potentially limited in AC hearts. However, emerging evidence suggests the presence of resident cardiac cell populations with ability to differentiate into fibroblasts, adipocytes or both. It is important to note that not a single cell population should be considered the only source to cardiac fibro-adiposis, as many populations can possibly overlap at different developmental and pathological stages. As discussed, the epicardium presents a strong candidate to fibro-fatty cellular differentiation. However, Isl1^+^ progenitors, which were suggested to originate adipocytes in AC, can also give rise to epicardium [87]. Additionally, FAPs and MSCs, which seem to present overlapping cell populations, can be derived via epicardial EMT [14, 72], and hence can act as intermediate multipotent mesenchymal cell populations (Fig. 2).

From a clinical perspective, current AC treatments are mainly directed towards relieving symptoms and halting disease progression to heart failure. These include antiarrhythmic drugs, implantable cardioverter defibrillators, catheter ablation, and optimally heart transplantation [17]. However, with the ongoing molecular understanding to AC pathogenesis, treatments targeted towards specific molecular pathways present promising therapeutic alternatives. Using a high-throughput screening approach, the WNT pathway activator SB216763 was identified as a novel candidate to prevent cardiac dysfunction in zebrafish and mouse models of AC [2, 12]. However, specifically related to fibro-fatty remodelling, difficulties with recapitulating this phenomenon in murine AC models has halted testing potential targeted therapies in a pre-clinical setting. However, one recent study showed that boosting levels of oxidized low-density lipoprotein (oxLDL) through high fat diet feeding in *Pkp2* heterozygous knockout mice that normally display no overt phenotype leads to sub-epicardial adipogenesis and ventricular systolic impairment [73]. Since AC patients with severe cardiac dysfunction and fibro-fatty tissue display high plasma levels of oxLDL, these mice could serve as suitable models to test the potential of targeting fibro-fatty tissue in vivo [73]. Additionally, advances in hiPSC technologies have allowed mimicking human AC pathogenesis and studying key pathological pathways of the disease in vitro. By generating patient-specific cardiac cultures, our group recently showed that siRNA-mediated epicardial targeting of transcription factor TFAP2A can reduce AC-induced EMT and fibro-fatty differentiation, which remains to be validated in a pre-clinical setting [43].

Owing to the sub-epicardial pre-dominance of fibro-fatty infiltrates in AC patients, therapies can potentially be conveniently directed towards the pericardial sac or the epicardial membrane using patches, catheters and slow-release hydrogels [59]. However, careful assessment to off-target effects, identifying optimal delivery systems, and whether a preventative therapy can be used at early stages of the disease, when fibro-fatty remodelling has not yet outspread in the heart, are yet to be studied. This will potentially help design better therapeutic options for patients with AC as well as other forms of cardiac disease.
